# Evaluation of the Bonding Shear Strength between Enamel and Dentin Feldspathic Porcelain and Two Different Monolithic Zirconia with Low and High Translucency

**DOI:** 10.1155/2024/5921637

**Published:** 2024-08-08

**Authors:** Amirhossein Fathi, Yeganeh Natanzian, Mahsa Ghorbani, Ramin Mosharraf

**Affiliations:** ^1^ Department of Prosthodontics Dental Materials Research Center Isfahan University of Medical Sciences, Isfahan, Iran; ^2^ Dental Students' Research Committee School of Dentistry Isfahan University of Medical Sciences, Isfahan, Iran; ^3^ School of Dentistry Mashhad University of Medical Sciences, Mashhad, Iran

## Abstract

**Introduction:**

The utilization of ceramics in the field of dentistry has seen a significant rise owing to their esthetic appeal and excellent functional properties. The use of ceramics in the field of dentistry has witnessed a notable surge, driven by their appealing esthetics and exceptional functional attributes. Zirconia, distinguished by its exceptional mechanical strength, plays a pivotal role in the fabrication of posterior crowns and bridges. Among zirconia variants, monolithic zirconia stands out, where the entire restoration is crafted from zirconia material. In parallel, feldspathic porcelain, chosen for its remarkable resemblance to natural tooth enamel, represents another significant ceramic type. This study aims to evaluate the shear bond strength (SBS) between two types of monolithic zirconia with two types of feldspathic porcelain.

**Methods and Materials:**

Forty-four monolithic zirconia veneered discs with feldspathic porcelain were subjected to SBS testing. The dimensions of the discs were 7 mm in diameter and 5 mm in height (3 mm of zirconia and 2 mm of porcelain). Subsequently, the specimens were subjected to a universal testing machine at a speed of 0.5 mm/min until failure occurred. The type of failure was examined using scanning electron microscopy. One-way analysis of variance (ANOVA), two-way ANOVA, Fisher's test, and multiple Tukey comparisons were used as statistical analyses.

**Results:**

The highest SBS was achieved by the high-translucency monolithic zirconia with enamel porcelain group (18.81 ± 3.18 MPa) and the high-translucency monolithic zirconia with dentin porcelain group (17.89 ± 2.75 MPa), followed by the low-translucency monolithic zirconia with dentin porcelain group (15.04 ± 2.24 MPa) and the low-translucency monolithic zirconia with enamel porcelain group (14.33 ± 2.00 MPa), respectively. Additionally, the most common type of failure pattern observed was mixed, followed by adhesive failure.

**Conclusion:**

The translucency of the porcelain did not significantly affect SBS, while the type of monolithic zirconia used had a significant impact. Furthermore, there was no discernible relationship between the four groups in terms of the distribution of failure patterns.

## 1. Introduction

The utilization of ceramics in the field of dentistry has seen a significant rise owing to their esthetic appeal and excellent functional properties. This upward trend can be attributed to the continuous improvements in ceramic materials and porcelain bonding systems [1]. Among the various types of porcelain used in dentistry, feldspathic porcelain has gained popularity. This is due to its high translucency and close resemblance to natural tooth enamel, making it a favored choice for various dental applications, especially for all-ceramic restorations [2]. However, despite the increasing use of these restorations, they still face certain limitations, including issues related to strength, translucency, how well they fit, and surface roughness [3].

Zirconia, introduced in dentistry as a core-supporting material in the early 1990s, has gained popularity due to its high strength. It is commonly used in posterior restorations and bridges [4]. Notably, patients who have received zirconia restorations exhibit significantly lower plaque index values compared to those who have been provided with traditional metal-ceramic restorations, suggesting easier long-term oral hygiene maintenance [5]. Monolithic zirconia restorations, crafted exclusively through CAD/CAM (computer-aided design and computer-aided manufacturing) technology, are entirely made of zirconia. These restorations boast high flexural strength, require less invasive dental preparation, reduce wear on opposing teeth, and necessitate less laboratory and chair time. As single-piece structures, they also avoid the risk of chipping [6, 7]. They are particularly recommended for patients with challenging occlusions, parafunctional habits, a history of fractures, or limited space for restorative materials [7, 8, 9, 10].

Previously, the primary drawback of monolithic zirconia was its poor esthetic quality due to lack of transparency [7, 11]. However, recent improvements in material composition, structural design, and fabrication methods have greatly enhanced their translucency, though this has come with a significant reduction in strength [9, 12, 13, 14].

Adding veneering porcelain to monolithic zirconia restorations is primarily done to improve esthetics. While monolithic zirconia offers adequate strength, enamel and dentin veneering porcelains can better replicate the natural look of the tooth [15]. This is particularly important in the anterior region, where esthetics are crucial. Furthermore, veneering porcelain is useful for precise occlusal adjustments. If the occlusion of the manufactured monolithic zirconia restoration is not ideal or if contact points are open, veneering porcelain allows for detailed adjustments to achieve optimal occlusal relationships and contact points [15].

The precise mechanism of porcelain bonding to zirconia is not yet fully understood, but micromechanical interactions are considered the primary factor [16]. The susceptibility to veneer chipping highlights the importance of customized recommendations from zirconia manufacturers for selecting the appropriate porcelain materials to optimize bonding with their specific zirconia substrates, emphasizing the crucial role of understanding material properties for correct clinical use [17, 18]. However, there is still a lack of comprehensive studies comparing the fracture strength of monolithic restorations to various types of veneered ones [2].

While many studies have explored the bond strength between monolithic zirconia and composite resins [19, 20, 21], and others have investigated the bond between feldspathic porcelain and zirconia cores to identify optimal materials and methods for enhancing the bond [22, 23, 24], there is a notable absence of research specifically examining the bond strength between monolithic zirconia and feldspathic porcelain. By addressing this gap, our research contributes to a more comprehensive understanding of the bonding behavior of monolithic zirconia with feldspathic porcelain, which is crucial for improving restorative dental materials and techniques. Therefore, this study aims to evaluate the shear bond strength (SBS) between two types of feldspathic porcelain (enamel and dentin) with two distinct models of monolithic zirconia (high and low).

## 2. Methods and Materials

Forty-four dental ceramic disks, with 7 mm diameter and 5 mm thickness, were evaluated (22 disks were fabricated from low-translucency monolithic zirconia, and 22 disks were constructed from high-translucency monolithic zirconia). The utilized formula for determining the sample size is as follows:

(1) $$\begin{array}{c} n=\frac{{\left(Z_{1-\frac{\alpha }{2}}+Z_{1-\beta }\right)}^{2}\sigma^{2}}{d^{2}}, \end{array}$$

where $Z_{1-\frac{\alpha }{2}}$ = 1.96, *Z*_1−*β*_ = 0.84, *α* = 0.05, 1 − *β* = 0.80, *n* = 11 for each group.

Based on the findings reported by Ahmadzade et al. [25], a discernible variance of 0.8 was observed between the groups. With an intended statistical power of 84%, the necessary sample size for all groups, while upholding a significance level of *α* = 0.05, was determined to be 44 specimens, with an allocation of 11 specimens to each group. Sample size calculations were performed using GPOWER v3.0.1 software (Dusseldorf, Germany).

The production process involved the utilization of a CAD–CAM device for precision Wieland milling. The specimens were meticulously processed in accordance with the precise protocols provided by the material manufacturer. This entailed subjecting the disks to the sintering procedure for 12 hr and subsequent sandblasting with 120 *µ*m aluminum oxide.

Afterward, each group of zirconia disks with different translucency was randomly divided into two subgroups using a simple randomization method. Subsequently, porcelain application (Noritake, Aichi, Japan) and firing at a final temperature of 930°C in an oven (Ivoclar, Schaan, Liechtenstein) were executed as per the manufacturer's recommendations.

**Figure 1:**
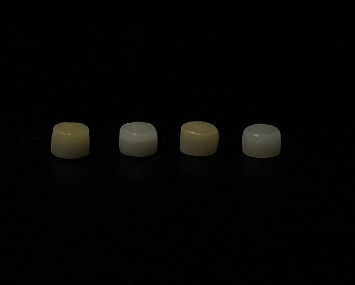
Monolithic zirconia discs veneered with feldspathic porcelain.

In the initial group, low-translucency monolithic zirconia (Ceramill zi, AmannGirrbach, Austria) was combined with enamel porcelain (ENLT). The second group paired low-translucency monolithic zirconia (Ceramill Zolid, AmannGirrbach, Austria) with dentin porcelain (DLT). The third group employed high-translucency monolithic zirconia (Ceramill Zolid ht+, AmannGirrbach, Austria) along with enamel porcelain (ENHT), whereas the fourth group employed high-translucency monolithic zirconia alongside dentin porcelain (DHT). Following these steps, measurements were performed using a gauge until the porcelain reached a thickness of 2 mm, after which the samples underwent glazing at a temperature of 910°C (Figure 1).

In the subsequent phase, the specimens were subjected to thermal cycling in a controlled environment. This involved cycling between temperatures of 5 and 55°C over a total of 5,000 cycles, approximating 1 year of clinical use. Each cycle included immersion in a bath for 30 s followed by a 10-s interval.

**Figure 2:**
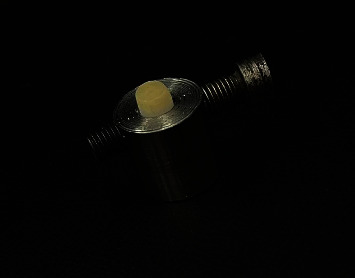
A disc mounted in a fixture made from stainless steel.

**Figure 3:**
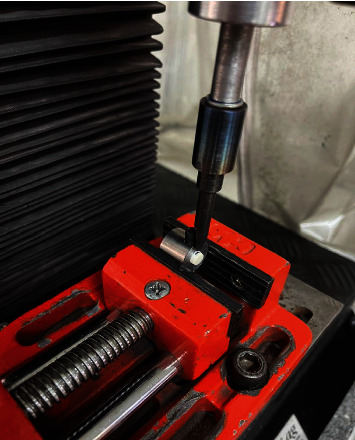
The method of applying force using a universal testing machine.

For the assessment of SBS, the samples were individually secured in stainless steel fixtures (Figure 2). Subsequently, the samples underwent SBS testing utilizing the Instron MTD-500 plus universal testing machine (SD Mechatronik, Westerham, Germany). A constant crosshead speed of 0.5 mm/min was maintained throughout the tests. Special care was taken to ensure that the applied force remained parallel to the contact interface between the zirconia and porcelain components (Figure 3). The force was gradually applied by the machine until a fracture occurred in each specimen. The resulting data were subsequently converted into megapascals (MPa) using the formula (shear bond = *N*/(38/465)).

Following the mechanical testing, the specimens underwent a cleaning process using ultrasonic equipment to eliminate any potential contaminants. The specimens were then subjected to microscopic examination at 400x magnification using an electron microscope (Leo/Ziess model1455vp, Oberkochen, Germany). The type of fracture observed was categorized into three distinct classifications:

1. Cohesive fracture occurring exclusively within the porcelain material.
2. Adhesive fracture localized at the interface between the porcelain and zirconia.
3. Mixed fracture, characterized by simultaneous damage within the porcelain and at the porcelain-zirconia interface.

### 2.1. Statistical Analysis

For statistical analysis and comparative assessment between the experimental groups, mean fracture diameters and maximum applied forces were calculated, and standard deviations were determined. One-way analysis of variance (ANOVA) was applied, followed by Tukey's multiple comparison post-hoc test to identify significant differences among the groups.

If abnormal distribution was confirmed by the Kolmogorov–Smirnov test, logarithmic transformation was applied to the data. Subsequently, after verifying the homogeneity of variances with Levene's test, the transformed data underwent statistical analysis using two-way ANOVA.

One-way ANOVA was initially applied to assess differences in mean SBSs across multiple groups (i.e., DHT, DLT, ENHT, and ENLT), and two-way ANOVA was employed to investigate the effects of two independent variables simultaneously: the type of porcelain (EN vs. D) and the type of monolithic zirconia (HT vs. LT). All statistical analyses were executed using the SPSS 26.0 (IBM, NY, USA), with a significance level set at 5% (*p*-value <0.05 was considered statistically significant).

## 3. Results

The mean SBSs were assessed retrospectively in various groups of monolithic zirconia specimens subjected to different veneering treatments. The average SBS was 75.2 ± 17.89 MPa in the DHT group. Moreover, for specimens in the DLT group, the average bond strength was measured at 15.04 ± 2.24 MPa. In the ENHT group, the bond strength was determined to be 18.81 ± 3.18 MPa. Lastly, in the ENLT, the mean bond strength was 14.33 ± 2.00 MPa (Table 1). The highest SBS is associated with the group of monolithic zirconia veneered with enamel porcelain, and then, in descending order, the groups of monolithic zirconia with high-translucency veneered with dentin porcelain, monolithic zirconia with low-translucency veneered with dentin porcelain, and finally monolithic zirconia with low-translucency veneered with enamel porcelain.

Given that the data from one of the four groups did not exhibit a normal distribution according to the Kolmogorov–Smirnov test, and after confirming homogeneity of variances using Levene's test (*p*-value = 0.810), the transformed data underwent statistical analysis using two-way ANOVA. The results showed that the effect of porcelain type on SBS was not significant (*p*-value = 0.997), but the effect of monolithic zirconia type was statistically significant (*p*-value <0.001). Furthermore, there was no significant interaction effect between the two independent variables (*p*-value = 0.308). In addition, one-way ANOVA was conducted to compare the four groups, revealing a significant difference among them (*p*-value <0.001). In addition, the Tukey test showed that there was no significant difference between the DHT and ENHT groups, as well as between the DLT and ENLT groups (Table 1).

**Figure 4:**
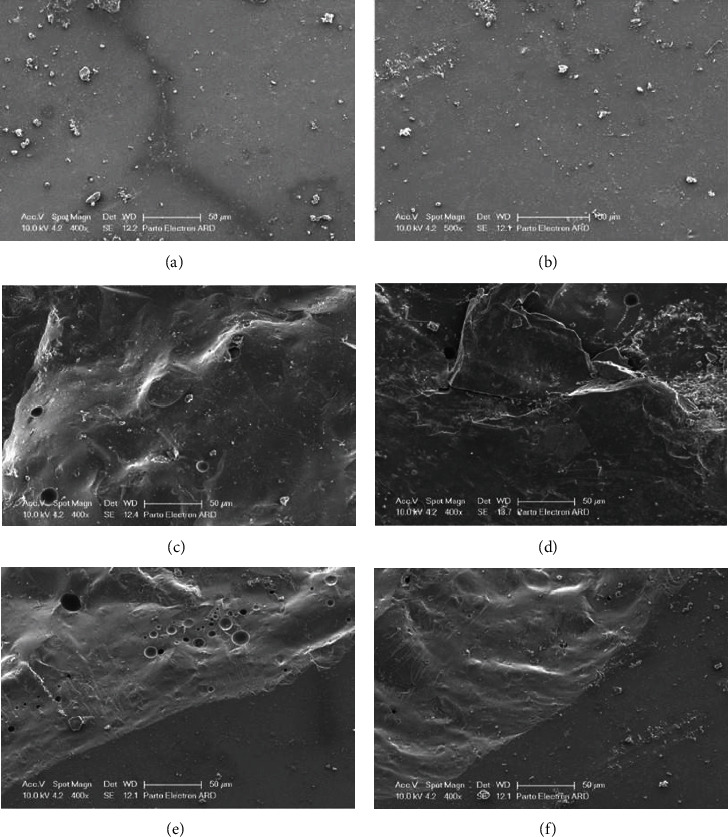
Various fracture patterns observed under the scanning electron microscope (SEM): (a and b) adhesive pattern, (c and d) cohesive pattern, and (e and f) mixed pattern.

In the DHT group, the most prevalent fracture pattern was of the mixed type. In the DLT group, the most common fracture patterns were a combination of adhesive and mixed types. Among the ENHT group, the predominant fracture pattern was the mixed type. Lastly, in the ENLT group, most fractures were of the adhesive type (Table 2). For comparing the distribution of fracture patterns among the four groups, the Fisher exact test showed that there was no statistically significant difference in the distribution of fracture patterns among the four groups (*p*-value = 0.449).  Figure 4 shows the different fracture patterns observed under the scanning electron microscope (SEM).

## 4. Discussion

According to the results of the present study, the type of feldspathic porcelain (enamel and dentin) did not have a significant effect on SBS, whereas the type of zirconia monolithic material significantly impacted the SBS. Notably, the highest SBS was observed with high-translucency zirconia monolithic veneered with feldspathic porcelain.

The significant effect of the type of zirconia on bond strength can be attributed to several factors related to the material properties of high-translucency zirconia. High-translucency zirconia is characterized by its microstructure and the grain size of the cubic crystals used to enhance its optical properties. The cubic crystals in high-translucency zirconia are isotropic, which means they have uniform properties in all directions [8, 26]. This isotropy results in a more homogenous composition that can improve bonding properties. A finer microstructure provides a more uniform and consistent surface for feldspathic porcelain to adhere to and leads to better wetting by the porcelain, enhancing mechanical interlocking and chemical bonding at the interface [27].

While no studies have assessed the bond between monolithic zirconia and feldspathic porcelain in the literature, it is important to note that studies regarding the bond strength of porcelain to zirconia cores are present. According to various clinical studies, most failures in zirconia core-porcelain restorations occur at the adhesion surface. The reasons for these failures may be due to material composition, properties, firing temperature, cooling rate, operator skill, porosity, and the manufacturing process, all of which can impact the quality and strength of the bond between the core and veneer materials [28]. To meet the requirements of ISO 9693, the average bond strength and the onset of fracture should be greater than 25 MPa [29]. We decided to use the SBS test method due to its ease of sample preparation, simple testing protocol, and the ability to rank different products based on bond strength values. However, the SBS test has drawbacks such as high standard deviation and the occurrence of nonuniform surfaces [30].

There have been limited previous studies measuring and comparing SBS between zirconia and various porcelain veneer types. In a study by Diniz et al. [31] that examined the SBS and failure of four types of porcelain veneers with tetragonal zirconia, it was shown that the SBS between zirconia and porcelain was significantly lower compared to metal and porcelain. The study found no significant difference between the three porcelain groups (IPS, Ceramco, Zirconzanh). In addition, the SBS values obtained for these three groups were similar to the average SBS in our study. Additionally, similar to the present study, all types of failures were observed.

Rismanchian et al. [32] study evaluated SBS between two types of zirconia (Biodenta and Cercon) and their respective dentin porcelains, showing no significant difference in SBS at the core-veneer interface between the two types of veneered zirconia with porcelain. The average SBS values obtained (22.28 and 19.27 MPa) were significantly higher than those in our study. However, the type of failure differed in the Biodenta system (adhesive failure) and the Cercon system (mixed failure), while in our study, there was no significant correlation between the four groups, and the highest type of failure was mixed. The reason for the difference between the two studies' results can be due to the utilization of different materials.

Moses et al. [23] study compared SBS at the zirconia-veneer interface between porcelain feldspathic and lithium disilicate with zirconia cores and found that the average SBS between porcelain feldspathic and zirconia was significantly lower than the values obtained in our study. Therefore, the results of this study contradict the findings of our study.

Moreover, similar to the present study, Hu et al. [33] study also showed that SBS at the zirconia-veneer interface is influenced by the selected zirconia system and that the thickness of the veneering ceramic has an impact on SBS.

The findings of this study have important clinical implications for the selection of porcelain materials in conjunction with different monolithic zirconia systems. Given that the type of zirconia significantly affects the SBS, with high-translucency zirconia demonstrating superior bonding to feldspathic porcelain, clinicians should consider this when planning restorative treatments. When selecting the type of feldspathic porcelain to use with high-translucency zirconia, it is important for clinicians to evaluate the specific needs of each case. While the type of feldspathic porcelain (enamel and dentin) did not significantly affect the bond strength in this study, the esthetic requirements, functional demands, and patient-specific factors should guide the choice of porcelain material.

For further studies, the authors suggest that the effect of different porcelain veneering techniques on monolithic zirconia cores and their impact on SBS need to be evaluated. In addition, determining various surface preparation techniques and their effect on SBS between monolithic zirconia and feldspathic porcelain can be lucrative.

One limitation of the present study is the challenge of replicating the oral environment accurately. Dental materials are tested in vitro, often using simplified models or artificial conditions that do not fully mimic the complex and dynamic conditions found in the human mouth. This limitation can affect the reliability of the results and their ability to predict how materials will perform in actual clinical situations. Additionally, long-term studies are often difficult to conduct in vitro, making it challenging to assess the durability and stability of dental materials over extended periods, which is crucial for evaluating their clinical effectiveness.

## 5. Conclusions

The type of feldspathic porcelain veneer does not significantly affect SBS, but the type of monolithic zirconia has a noticeable impact. Monolithic zirconia with high translucency demonstrates higher bond strength, and overall, among the four sample groups tested in our study, the group of high-translucency monolithic zirconia veneered with feldspathic porcelain exhibits the highest SBS. The most common type of failure among the four groups is a mixed failure, accounting for 50%, followed by adhesive failure at 32%. Overall, there is no significant correlation between the distribution of fracture patterns among the four groups. Due to the lower SBS values between monolithic zirconia and feldspathic porcelain compared to the minimum threshold specified in ISO 9693, it is not advisable to veneer monolithic zirconia with feldspathic porcelain.

## Figures and Tables

**Table 1:** The mean shear bond strengths (MPa) and pairwise comparison across groups.

Porcelain	Zirconia	Mean	Number	Standard deviation	Lowest value	Highest value	Pairwise *P*-value			
							DHT	DLT	ENHT	ENLT
Dentin	HT	17.89	11	2.75	14.97	22.27	—	—	—	—
	LT	15.04	11	2.24	11.77	18.25	0.050^*∗*^	—	—	—

Enamel	HT	18.81	11	3.18	14.13	24.90	0.886	0.008^*∗*^	—	—
	LT	14.33	11	2.00	10.90	17.96	0.008^*∗*^	0.884	0.001^*∗*^	—

HT, high translucency; LT, low translucency.  ^*∗*^Indicates a significant difference as result of Tukey test.

**Table 2:** Distribution of fracture types across four groups.

	Groups				Total	*P*-value
	DHT	DLT	ENHT	ENLT		
Fracture types						
AD						0.449
N	1	5	3	5	14	
%	9.1	45.45	27.3	45.45	31.8	
COH						
N	2	1	3	2	8	
%	18.2	9.1	27.3	18.2	18.2	
MIX						
N	8	5	5	4	22	
%	72.7	45.45	45.45	36.4	50	
Total						
N	11	11	11	11	44	—
%	100	100	100	100	100	

## Data Availability

The data supporting the results of this study are available upon reasonable request from the corresponding author.
